# Keeping kids in school: modelling school-based testing and quarantine strategies during the COVID-19 pandemic in Australia

**DOI:** 10.3389/fpubh.2023.1150810

**Published:** 2023-06-02

**Authors:** Romesh G. Abeysuriya, Rachel Sacks-Davis, Katherine Heath, Dominic Delport, Fiona M. Russell, Margie Danchin, Margaret Hellard, Jodie McVernon, Nick Scott

**Affiliations:** ^1^Disease Elimination Program, Burnet Institute, Melbourne, VIC, Australia; ^2^Department of Epidemiology and Preventive Medicine, Monash University, Melbourne, VIC, Australia; ^3^Murdoch Children’s Research Institute, Parkville, VIC, Australia; ^4^Department of Paediatrics, The University of Melbourne, Parkville, VIC, Australia; ^5^The Royal Children’s Hospital, Melbourne, VIC, Australia; ^6^School of Population and Global Health, The University of Melbourne, Parkville, VIC, Australia; ^7^Department of Infectious Diseases, The Alfred and Monash University, Melbourne, VIC, Australia; ^8^Department of Infectious Diseases, The University of Melbourne at the Peter Doherty Institute for Infection and Immunity, Melbourne, VIC, Australia; ^9^Victorian Infectious Diseases Epidemiology Unit, The Royal Melbourne Hospital at the Peter Doherty Institute for Infection and Immunity, Melbourne, VIC, Australia

**Keywords:** COVID-19, outbreak, rapid antigen test, agent-based model, school, surveillance, quarantine

## Abstract

**Background:**

In 2021, the Australian Government Department of Health commissioned a consortium of modelling groups to generate evidence assisting the transition from a goal of no community COVID-19 transmission to ‘living with COVID-19’, with adverse health and social consequences limited by vaccination and other measures. Due to the extended school closures over 2020–21, maximizing face-to-face teaching was a major objective during this transition. The consortium was tasked with informing school surveillance and contact management strategies to minimize infections and support this goal.

**Methods:**

Outcomes considered were infections and days of face-to-face teaching lost in the 45 days following an outbreak within an otherwise COVID-naïve school setting. A stochastic agent-based model of COVID-19 transmission was used to evaluate a ‘test-to-stay’ strategy using daily rapid antigen tests (RATs) for close contacts of a case for 7 days compared with home quarantine; and an asymptomatic surveillance strategy involving twice-weekly screening of all students and/or teachers using RATs.

**Findings:**

Test-to-stay had similar effectiveness for reducing school infections as extended home quarantine, without the associated days of face-to-face teaching lost. Asymptomatic screening was beneficial in reducing both infections and days of face-to-face teaching lost and was most beneficial when community prevalence was high.

**Interpretation:**

Use of RATs in school settings for surveillance and contact management can help to maximize face-to-face teaching and minimize outbreaks. This evidence supported the implementation of surveillance testing in schools in several Australian jurisdictions from January 2022.

## Introduction

Until mid-2021, Australia endeavored to strongly suppress community SARS-CoV-2 transmission by limiting incursions with tight border controls and containing outbreaks with contact tracing and strict community restrictions, including extended lockdowns in some jurisdictions. However, the rollout of COVID-19 vaccines meant that Australia could consider alternate approaches to managing COVID-19 without either the social and economic impact associated with containing outbreaks, or the dire health outcomes associated with community transmission prior to vaccines being available. In late 2021, the Commonwealth Government commissioned the Doherty Institute to lead a consortium of modelling groups to support development of the National Plan to transition Australia’s COVID-19 response (1).

While Australia pursued a goal of zero community SARS-CoV-2 transmission (2020–2021), schools were often closed as part of broader lockdown measures. When schools were open, outbreaks in most jurisdictions were managed by reactive closures and targeted periods of quarantine. This typically involved a school closing for cleaning following a positive case (often two to 3 days), followed by a 14-day quarantine for all close contacts and their households. Specific school outbreak strategies differed between states and territories but were generally commensurate with other community restrictions, and were successful in reducing the size of school outbreaks (2). However, due to concern about the potential health and psychosocial impacts of these public health responses on children (3), maximizing in-person learning was seen as a national priority.

With Australia’s transition to “living with COVID-19” and increased community transmission, the rates of incursions into schools were anticipated to be higher than previously experienced (4). In this context, a quarantine-based approach in schools was recognized to be unsustainable and inconsistent with the national priority of maximizing face-to-face teaching. Hence there was a need for different approaches to managing cases and contacts in schools and keeping schools open safely.

This commissioned work was developed through a participatory process with the Commonwealth Departments of Health and Education. Its agreed focus was to support face-to-face teaching through identification of strategies that would minimize importation and transmission of infections in the school environment. Given this objective, infections and days of face-to-face teaching lost following an imported case were defined as the key outcomes of interest. The specific aims of this study were to evaluate the potential impacts of two strategies identified in consultation: the use of rapid antigen tests (RATs) for asymptomatic screening, or as an alternative risk mitigation to home quarantine of close contacts, based on their effective implementation in other country settings (5–7). The scope of enquiry was restricted to transmission risks anticipated in primary and secondary day schools, and analyses were conducted prior to the emergence of the Omicron variant.

## Methods

### Model overview

An established agent-based microsimulation model, *Covasim* (8), was used to simulate outbreaks in school settings. The model is open-source and available online (9) and has previously been used to model epidemic waves and response strategies in Australia (10–12). Additional model details are provided in the Supplementary material. The code for the simulations and analysis presented in this study is available at https://github.com/optimamodel/covasim_aus_schools.

To address the specific questions of this study, we implemented a more detailed model of school contact networks than those provided by *Covasim*, based on the structure of the Australian school system. Aside from differences in age and vaccine eligibility, there are important differences in social and mixing structure between primary and secondary schools; hence these two settings were modelled separately. Schools were characterized by three types of interaction – transmission within classrooms involving students and teachers, transmission outside of classrooms between students (e.g., during breaks), and transmission outside of classrooms between teachers (e.g., in staff common rooms).

### Primary schools

**Figure 1:**
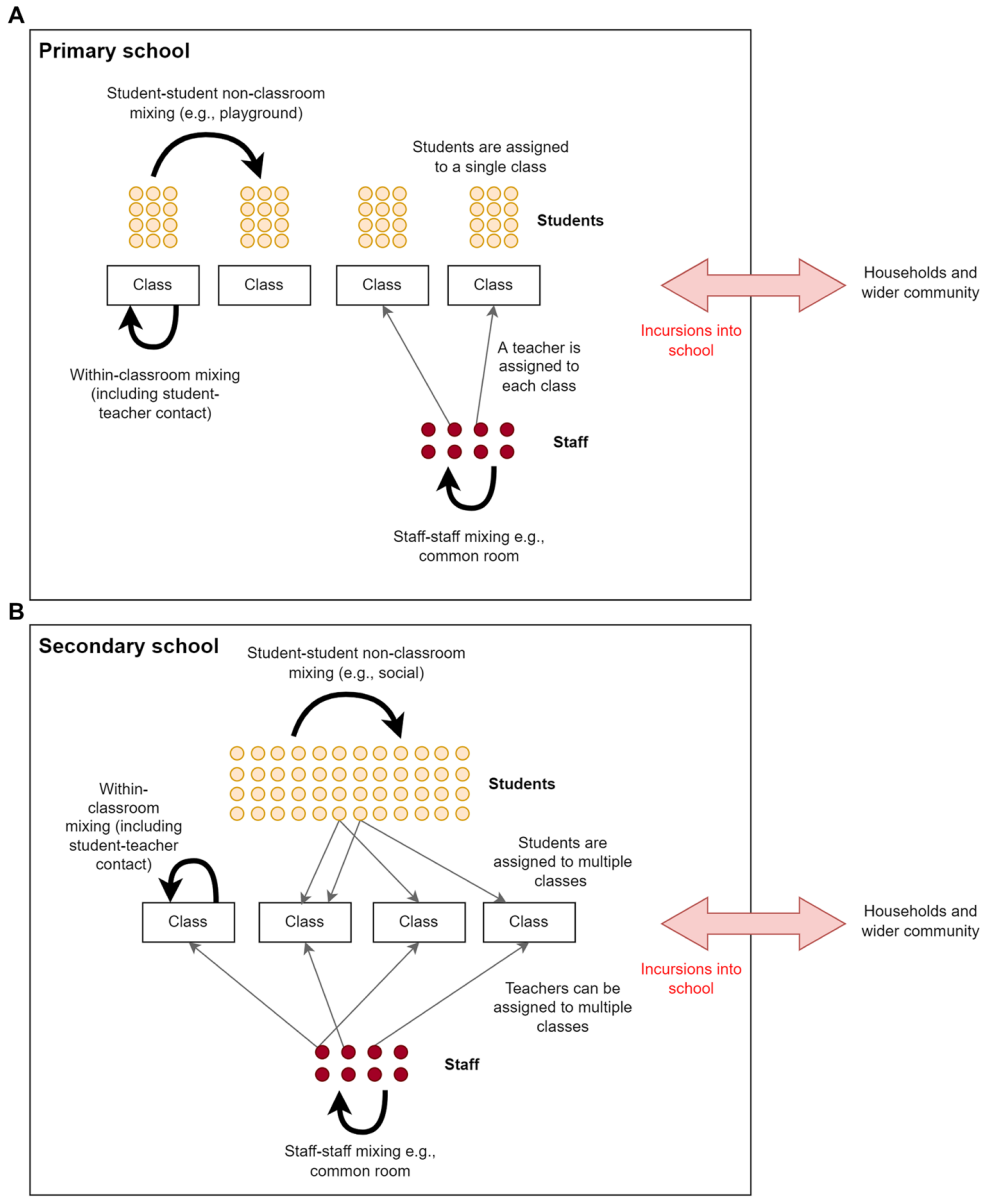
Contact networks within schools in the model for a) primary schools, and b) secondary schools. Schools included student–student classroom contacts, student–student non-classroom contacts, teacher-teacher contacts and teacher-student contacts. Primary schools were modelled as a collection of classrooms, where students of the same age are assigned a teacher. Secondary school students and teachers have more contacts than primary school students and teachers because they attend multiple classes.

In Australia, primary school students are typically assigned to a single class for all lessons. Primary schools were therefore modelled as a collection of classrooms, aggregated into schools. To construct the primary school contact network, children from ages 5–11 were assigned to schools. Within each school, students were assigned to classrooms with others of the same age, and each classroom had an assigned teacher (randomly selected from the working-age population, age 18–65). Each classroom was a fully connected cluster, with transmission possible between any pair of individuals within the same classroom. The classroom contact network was therefore highly clustered (Figure 1A). Non-classroom mixing was incorporated by assigning each student additional contacts randomly selected from the entire school.

### Secondary schools

Secondary school students in Australia are typically grouped into separate classes for each subject and have around 4–6 classes per day. To simplify the implementation and parameterization of the model, instead of modelling each classroom explicitly, students were assigned a number of random contacts amongst other students of the same age, with the number of contacts reflecting the average number of different students they would encounter each day. Each student was then randomly assigned a number of contacts with staff members reflecting the typical number of classes per day, and a number of contacts with other students randomly selected from the entire school to account for non-classroom mixing. Compared to primary schools, there is considerably more mixing between students and teachers within classrooms, and classroom contacts are much less clustered (Figure 1B).

### Disease transmission

Transmission in the model has a probability of occurring each time a susceptible individual is in contact with an infectious individual through one of their contact networks. The overall transmission probability per contact per day was calibrated based on the Delta variant epidemic wave in Melbourne over the July–September 2021 period (12). For individual contacts, this transmission risk was further weighted according to the setting of the contact (e.g., classroom, home), the time-varying viral load of the person infected, whether or not they have symptoms (based on an age-specific probability of being symptomatic), and an age-specific disease susceptibility (Table 1).

**Table 1:** Model parameters related to schools.

Parameter area	Estimate	Source
Primary school		
Average school size	298	Number of primary students (2,268,891 full time + part time in 2020; ABS (13) Table 42b) divided by number of Primary + Primary/secondary schools (6249 + 1363 in 2020; ABS (13) Table 35b).
Average class size	22	Average class size of primary schools. Victorian government (14), with class sizes sampled from their distribution in analyses.
Average number of student–student non-classroom contacts per day, per student	2	Assumption; tested in sensitivity analysis. This impacts the efficacy of test-to-stay of class contacts verses close contacts or entire school.
Average number of teacher-teacher contacts per day, per teacher	5	Assumption.
Vaccination coverage	0%	Vaccines for under-12 children were not authorized at the time of analysis (a sensitivity analysis including vaccination in primary school children is provided in the Supplementary material).
Secondary school
Average students per school	622	Number of secondary students (1,738,083 full time + part time in 2020; ABS (13) Table 42b) divided by number of Secondary + Primary/secondary schools (1433 + 1363 in 2020; ABS (13) Table 35b)
Average teacher/student ratio	12	ABS data (13). Suggesting secondary schools have on average 12.1 students to one teacher.
Average number of student–student classroom contacts per day	44	Average class size in secondary school of 22 ((15); page 354), assuming two unique classrooms of contacts per student per day.
Average number of student–student non-classroom contacts per day	5	Assumption; tested in sensitivity analysis. This impacts the efficacy of test-to-stay of class contacts verses close contacts or entire school.
Average number of teacher-teacher contacts per day	5	Assumption.
Average number of teacher-student contacts per day, per student	6	Assumes students have six classes per day
Vaccine coverage	80%	Assumed peak coverage level, based on expected vaccine uptake at the time of analysis.
Probability of transmission per contact per day (without vaccines or NPIs)		
Student–student (primary classroom)	0.25	Delphi process; Scott et al. (10) Measured as relative to household transmission per contact - e.g. a typical day’s worth of contact in school is 75% less likely to result in transmission than a typical day’s worth of contact at home. Non-classroom primary school contacts equivalent to outdoor contacts; secondary school classroom contacts halved to account for shorter interactions. Note: these are not attack rates, and all transmission probabilities are scaled by an overall calibration parameter, as well as age-specific susceptibility, vaccine status, and NPIs in place. Attack rates also depend on frequency and number of contacts. All transmission probabilities were varied in sensitivity analyses when NPI efficacy is tested.
Student–student (primary non-classroom)	0.03	
Student–student (secondary class contact)	0.12	
Student–student (secondary close/social contact)	0.12	
Teacher-teacher	0.25	Assumption that transmission risks in schools are equivalent for all types of contacts. Note that the model has independent parameters to account for differences in susceptibility by age
Teacher-student (primary)	0.25	
Teacher-student (secondary)	0.12	
Age-susceptibility (relative to 20–49 year old)		
Age 0–4	0.349	Derived from Davies et al. (16)
Age 5–9	0.423	
Age 10–14	0.495	
Age 15–19	0.599	
Age 20–24	0.846	
Age 24–29	1	
Probability of being symptomatic		
Age 0–9	0.28	Davies et al. (16)
Age 10–19	0.20	
Age 20–29	0.26	
Rapid antigen testing (RAT)		
Sensitivity	0.773	Muhi et al. (17) Lower bound selected to account for inconsistent self-use. Note that PCR is modelled as having 87% sensitivity in real world use (systematic review Arevalo-Rodriguez et al. (18))

### Symptomatic testing probability (COVID-19 cases)

All people with severe disease were assumed to be tested. For people with mild symptoms, the model included a per-day probability of seeking a test, which was necessary for the first case to be diagnosed when surveillance was not in place (noting that the first case to be detected may be a household member of a student at the school, which would trigger contact tracing for the student). The symptomatic testing model calibration process estimated that people with mild symptoms who were not identified through contact tracing would seek testing during their symptomatic period with a per-day testing probability of 11% (varied in a sensitivity analysis).

### Vaccination

All baseline scenarios were run with 80% COVID-19 vaccination coverage in ages 12+ and 100% vaccination coverage in teachers, reflecting likely uptake of the vaccine at the time of analysis, the likelihood of vaccines becoming mandatory for teachers, and the fact that vaccines for ages 5–11 were yet to be approved for use in Australia at the time of reporting. However, sensitivity analyses were run to investigate the extent to which secondary schools in the baseline scenarios already benefited from vaccination and to assess the benefits of vaccines for primary school students if they became available (provided in the Supplementary material). Vaccine parameters were based on efficacy estimates against the Delta variant available at the time of analysis (19, 20) (see Supplementary material).

### School testing and tracing strategies

Two overarching strategies for incorporating RATs into schools were considered. The first was asymptomatic surveillance, where students were required to take RATs regularly regardless of any symptoms. The specific implementations considered were: no surveillance testing; twice weekly teacher testing with RATs; and twice weekly student testing with RATs. These scenarios were considered with and without contact tracing in place.

The second strategy considered was a “test-to-stay” scheme in which contacts of diagnosed cases performed daily RATs instead of being quarantined. The specific implementations of the test-to-stay scheme were: no contact tracing (neither testing nor isolation for contacts); 7 days quarantine of classroom contacts with/without daily RAT; daily RAT of classroom contacts who are permitted to remain at school so long as they test negative (“test-to-stay”); and entire school test-to-stay with daily RAT after initial case detection.

The scenario with both seven-day quarantine and daily testing for 7 days enables assessment of the benefit of quarantine incremental to test-to-stay, controlling for differences in case ascertainment. Contact tracing scenarios were based around classroom contacts rather than all contacts, as classroom contacts were deemed more practical to identify in response actions. Scenarios were also examined with both asymptomatic surveillance and test-to-stay to assess the incremental impact of surveillance strategies when combined with contact tracing.

In all scenarios, students or teachers diagnosed with COVID-19 were assumed to be removed from the school and required to isolate until no longer infectious.

### Model simulations and outcomes

The model was initialized with a single infection allocated randomly within a school. The model was then run for 45 days to allow sufficient time for the outbreak to grow within the school, while limiting the impact of broader community transmission on within-school dynamics. The number of cumulative infections in students or teachers attending the school were recorded. Infections were used as the primary outcome measure as opposed to diagnosed cases to avoid bias when comparing strategies with different testing rates. Importantly, although we report outcomes after 45 days, in cases where there is substantial transmission the outbreak is likely to be ongoing, and further transmission would take place after the simulation timeframe. In these cases, the cumulative number of infections after 45 days serves as a proxy measure for the growth rate of the outbreak. We elected not report the basic reproduction number $R_{0}$ because the short time window, small size, and wide range of stochastic outcomes makes this metric difficult to interpret for the outbreaks modelled in this study.

For each scenario, the simulation was repeated 1000 times and reported outcomes are based on the distributions of (1) secondary infections occurring in the same school; and (2) days of face-to-face teaching lost. Days of face-to-face teaching lost are calculated for the school as the total student-days spent in isolation or quarantine as a result of a school’s testing and quarantine policy over the 45-day period. A day of face-to-face teaching lost is accrued for each student, for each day that they are unable to attend school, and is therefore independent of the structure of the school.

### Sensitivity analyses

To enable the analysis to be applied across a wide range of contexts, sensitivity analyses were performed to examine how outcomes varied with different assumptions or inputs. The parameters that were varied were: school incursion rates (the model was initialized with one, two, or three simultaneous incursions as a proxy for community prevalence, where settings with high prevalence are more likely to have simultaneous or otherwise overlapping outbreaks due to high incursion rates from the community); and compliance with test-to-stay (also an equivalent sensitivity analysis for lower test sensitivity) ranging from 0 to 100%.

A number of other parameters were also varied, including vaccine coverage, non-pharmaceutical interventions, frequency of surveillance testing, number of non-classroom contacts, and symptomatic testing rate. These are provided in the Supplementary material.

### Total days of face-to-face teaching gained

The modelled scenarios provide estimates of the days of face-to-face teaching lost per incursion. The total number of days lost or gained can be estimated based on the number of school incursions that take place. An example of how this calculation could be performed is provided in the Supplementary material.

## Results

In this section, we simulate incursions in primary and secondary schools following an incursion event, and report on the number of downstream infections and days of face-to-face teaching lost under a range of control strategies. The outcomes described here are specific to the parameter values and assumptions outlined in the previous section, and therefore general trends should be considered for policy rather than the specific quantitative values.

### Surveillance strategies, without contact tracing/quarantine

**Figure 2:**
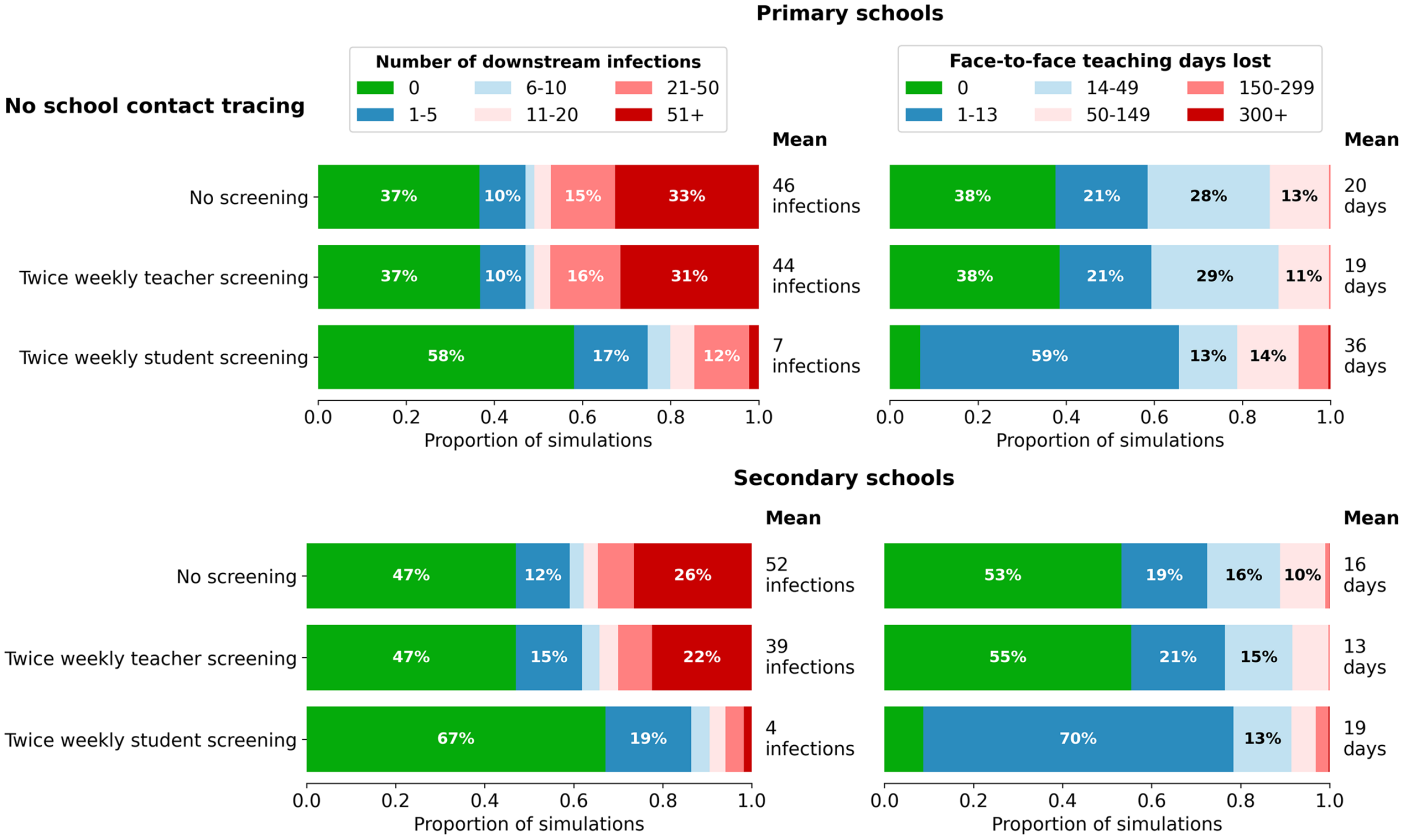
Impact of surveillance strategies on the distribution of outcomes for cumulative infections **(left)** and days of face-to-face teaching lost **(right)** in a single school following a single incursion. Outcomes are from 1,000 model simulations run for 45 days following first diagnosis. Scenarios assume no contact tracing or quarantine (only isolation for positive cases that are detected) and from top to bottom are based on: no screening; twice weekly testing of teachers with rapid antigen tests; twice weekly testing of students with rapid antigen tests.

We first examined the impact of surveillance testing, in the absence of contact tracing. In many simulations, the initial incursion led to less than five downstream infections, and of these, the majority had no onwards transmission at all (Figure 2).

Twice weekly screening of teachers had minimal impact on reducing infections in primary schools, and only a marginal impact in secondary schools. Twice weekly screening of students led to earlier detection of an incursion and increased the chances of an incursion leading to no secondary infections in both primary and secondary schools. Screening of students slightly increased the *mean* days of face-to-face teaching lost compared with no screening and no contact tracing due to the detection of asymptomatic cases. However, the average masks the fact that this scenario resulted in a 20% increase in the proportion of incursions where transmission was effectively averted by early detection, a marked reduction in outbreaks of size 20 or more, and a reduction in the proportion of simulations with 14 or more days of face-to-face teaching lost.

**Figure 3:**
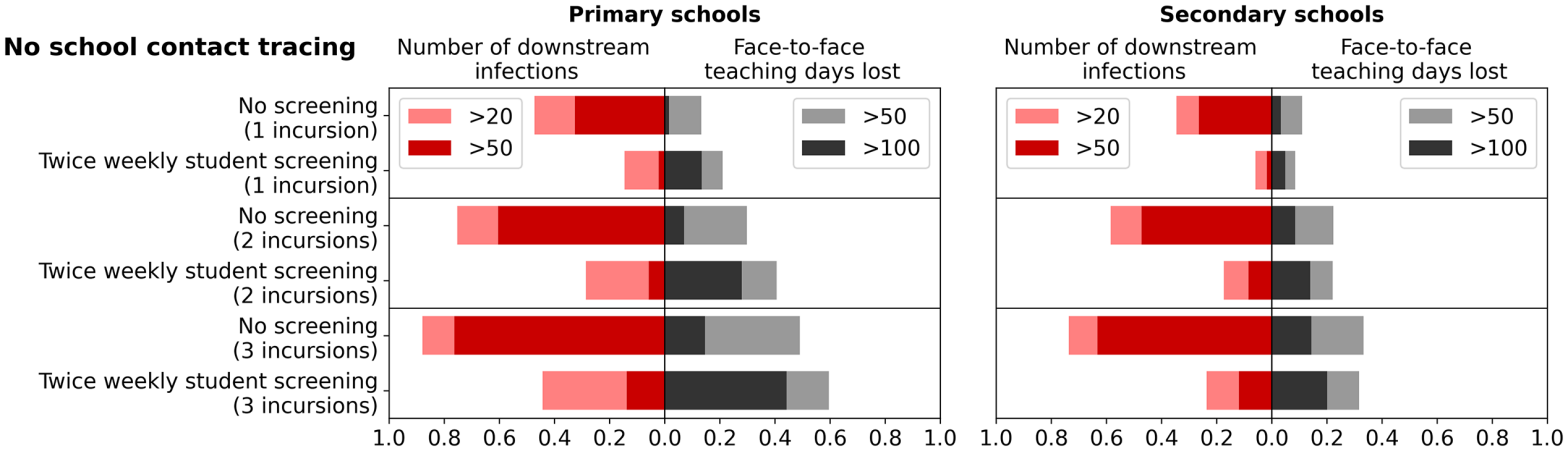
Impact of multiple incursions on the benefits of surveillance testing. Left bars: the percentage of simulations with more than 20 or 50 cumulative infections after 45 days of first diagnosis, for different surveillance strategies and number of initial incursions. Right bars: the percentage of simulations with more than 50 or 100 days of face-to-face teaching lost in a single school following the incursions. Outcomes are from 1,000 model simulations run for 45 days following first diagnosis. Scenarios assume no contact tracing or quarantine (only isolation for positive cases that are detected) and compare no screening to twice weekly testing of students with rapid antigen tests.

Twice weekly screening of students had a greater impact on reducing secondary infections in schools as the number of incursions increased (Figure 3). With increased numbers of incursions, days of face-to-face teaching lost in secondary schools remained similar with or without student screening. In primary schools, days of face-to-face teaching lost slightly increased with the screening in place regardless of number of incursions. Overall, as the number of incursions increased, the incremental benefits for reduced secondary infections were far greater than the increase in days of face-to-face teaching lost.

### Contact tracing and quarantine strategies: “test-to-stay”

**Figure 4:**
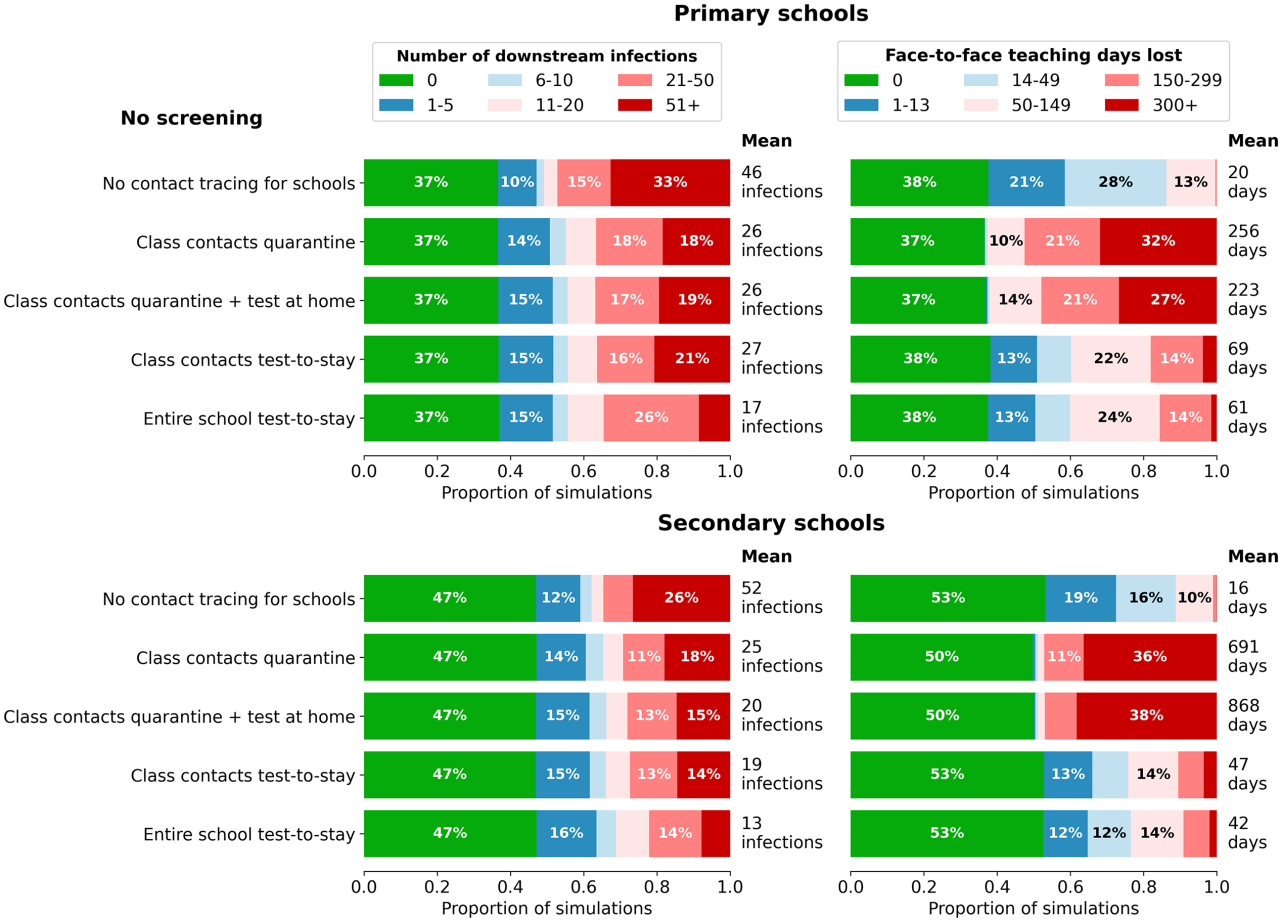
Impact of contact tracing and quarantine strategies on the distribution of outcomes for cumulative infections **(left)** and days of face-to-face teaching lost **(right)** in a single school following a single incursion. Outcomes are from 1,000 model simulations run for 45 days following first diagnosis. Scenarios top to bottom: no contact tracing; class contacts have 7-day quarantine without/with testing; class contacts test-to-stay with rapid antigen tests; entire schools test-to-stay with rapid antigen testing. Top: Primary schools; bottom: Secondary schools.

We next examined contact tracing and the impact of testing contacts compared to quarantining contacts, in the absence of surveillance testing. Quarantining classroom contacts of identified cases considerably decreases the mean size of outbreaks after 45 days – from 46 cases to 26 cases in primary schools, and 52 cases to 25 cases in secondary schools (Figure 4). However, this comes at the expense of a large number of face-to-face teaching days lost per incursion – an average of 256 days in primary schools, and almost 700 days in secondary schools.

**Figure 5:**
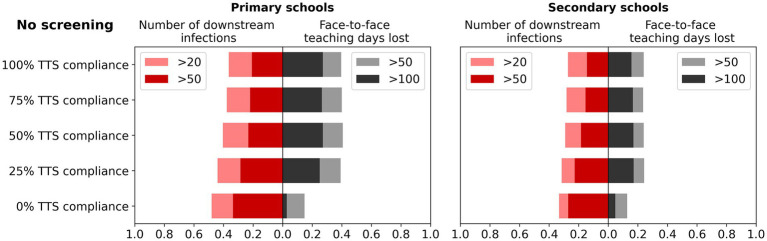
Impact of compliance on the effectiveness of a test-to-stay (TTS) strategy. Left bars: the percentage of simulations with more than 20 or 50 cumulative infections after 45 days of first diagnosis, for different TTS compliance. Right bars: the percentage of simulations with more than 50 or 100 days of face-to-face teaching lost in a single school following the incursion. Outcomes are from 1,000 model simulations run for 45 days following first diagnosis.

Test-to-stay of classroom contacts had approximately equivalent impacts on transmission as seven-day quarantine of classroom contacts in both primary and secondary schools, but without the associated face-to-face teaching days lost (Figure 4). Increased case ascertainment resulted in slightly more days of face-to-face teaching lost compared to the baseline no contact tracing scenario, but as with surveillance there was a marked reduction in the proportion of incursion leading to outbreaks of 20 or more. The effectiveness of test-to-stay decreased with low compliance, but conversely, there were diminishing returns at high levels of compliance (Figure 5).

The incremental benefit of testing all school contacts in addition to classroom contacts was small.

### Surveillance strategies combined with contact tracing/quarantine

Finally, we examined the impact of surveillance testing, when combined with contact tracing and a test-to-stay strategy. Twice weekly screening of students had incremental benefits in terms of reduced infections and reduced face-to-face teaching days lost (Supplementary material; Figure S1), even when test-to-stay was in place. By detecting and removing cases earlier, student screening reduces the number of downstream infections following an incursion, the likely outbreak size, and the average days of face-to-face teaching lost per incursion. In particular, screening resulted in a considerable reduction in the proportion of simulations where more than 150 days were lost.

### Summary of trends for surveillance and contact tracing strategies

The change in infections and days of face-to-face teaching lost for each of the control strategies and settings compared to no intervention is summarized in Table 2.

**Table 2:** Model-estimated percentage change in mean number of infections and days of face-to-face teaching lost in primary and secondary schools compared to no intervention, for the scenarios shown in Figures 2, 4.

Intervention	Primary schools		Secondary schools	
	Mean infections	Days lost	Mean infections	Days lost
Screening interventions				
Twice weekly teacher screening	−4% (−12,0)	−5% (−12,1)	−25% (−34,-17)	−19% (−31,-12)
Twice weekly student screening	−85% (−87,-83)	80% (65,96)	−92% (−94,-91)	19% (2,38)
Tracing interventions				
Class contacts quarantine	−43% (−47,-42)	1180% (1119,1257)	−52% (−55,-50)	4219% (3932,4548)
Class contacts quarantine + test at home	−43% (−46,-40)	1015% (963,1086)	−62% (−65,-60)	5325% (4923,5774)
Class contacts test-to-stay	−41% (−45,-39)	245% (229,269)	−63% (−65,-60)	194% (170,221)
Entire school test-to-stay	−63% (−66,-61)	205% (188,223)	−75% (−77,-74)	163% (140,184)

	No change		Moderate reduction		Large reduction		Moderate increase		Large increase

Results are presented as the percentage change relative to the no intervention scenario (95% confidence interval). Confidence intervals were calculated using the percentile bootstrap method with 1,000 replicates.

## Discussion

This study used an agent-based model to assess the effectiveness of a variety of school-based surveillance, contact tracing and quarantine strategies to reduce outbreaks and transmission in schools, and maximize face-to-face teaching. We found that twice-weekly surveillance testing of students markedly reduced outbreak risk by enabling early detection of incursions, and that a ‘test-to-stay’ contact tracing strategy could achieve equivalent outbreak containment to home quarantine, without the associated loss of face-to-face teaching days. This was true for both primary and secondary schools.

School based surveillance testing considerably increased the proportion of simulations where an incursion resulted in no more than five downstream infections within 45 days. The incremental benefits of student surveillance testing were greater as the incursion rate increased, indicating that surveillance testing is expected to have maximum utility in areas with higher-than-average community transmission. Surveillance screening of students was also found to act synergistically with contact tracing and could be added on top of other policies such as test-to-stay to further reduce infections and in-person teaching days lost in areas at risk of outbreaks, but this would need to be balanced against the burden of testing for students and teachers.

The test-to-stay strategy outperformed a home quarantine strategy significantly in terms of maximizing face-to-face teaching. This is because a single infection in a class can result in more than 20 students losing 7 days each, and if transmission occurs to other classrooms (or in secondary school where students have multiple subjects) then quarantine requirements can be multiplicative. For test-to-stay to perform equivalently to home quarantine in terms of minimizing outbreak size, students must be compliant with testing requirements; however, we found that much of the benefits were still realized with only 50% compliance. There are also concerns about the reduced sensitivity of rapid antigen tests to detect infections in classroom contacts, but in this appears to be compensated for with the high frequency of testing. The test-to-stay strategy was assessed with an assumed 80% vaccination coverage among students 12+ years, which was based on expected vaccine uptake at the time of analysis. The actual coverage of vaccines in Australia reached 77 and 88% among people aged 12–15 and 16–19 years by 31 January 2022 (21), suggesting this was a reasonable approximation, and sensitivity analyses showed that it would still be effective at lower vaccine coverage (as might be the case in international settings). Overall, the key finding that test-based strategies could provide epidemiological outcomes equivalent to quarantine was consistent with international empirical studies and evaluations that have occurred since (6, 7, 22, 23).

The report from the commissioned work was delivered to the Australian Government in November 2021 and was used to inform policy development and school-based strategies for 2022. The Australian school year aligns with the calendar year, and schools take summer vacations with most reopening in late January. In November–December 2021 the Omicron variant spread throughout Australia with cases peaking in early/mid-January 2022, substantially increasing prevalence in the community ahead of school reopening. In January 2022, state and federal governments developed the National Framework for Managing COVID-19 in Schools and Early Childhood Education and Care (24) affirming the importance of keeping schools open without prescribing specific policies as to how this was to happen. Accordingly, each state implemented their own return-to-school policies, depending on their specific health and education systems and local state of the epidemic. In January 2022, two states, South Australia and Western Australia, adopted the test-to-stay strategy, while Australia’s two most populous states (NSW and Victoria) and those with the highest case incidence of SARS-CoV-2 implemented a twice-weekly screening program, and daily testing for 5 days for children in higher risk special school settings.

### Strengths

A key strength of the modelling was that it was embedded as a part of the policy-making process. Modelers were engaged from the beginning, which enabled a deeper understanding of the questions most relevant to governments, and analyses could be collaboratively designed to best answer them. Another strength was that modelling was used to quantify key outcomes that could not be measured through data analysis or other methods. For example, quantifying the days of face-to-face teaching gained by early detection and isolation of infected students and the subsequent prevention of onward transmission. Finally, the modelling formed just one component of broader advice to government, who were therefore able to incorporate modelling outcomes alongside other forms of evidence, including data analysis, feasibility, acceptability, and logistic issues.

### Limitations and future work

The findings presented are derived from an individual-based model, which is an imperfect representation of the real world with uncertainties in many parameters relating to disease progression and transmission. Model parameters were based on best-available data at the time of analysis.

The specificity of RATs has been measured in the range of 99.73–100% (17) and we therefore elected not to include false positive test results in this study. There would be additional days of face-to-face teaching lost due to false positive results associated with the surveillance testing and test-to-stay strategies. However, aside from the high test specificity limiting the number of false positives, we also note that many of the false positives arising from surveillance testing would occur in the absence of an active outbreak, and are therefore not captured within the scope of the simulations examined here.

Modelling was conducted based on the Delta variant. However, sensitivity analyses suggest that outbreak risks and days of face-to-face teaching lost following an incursion are greater with a more infectious variant. This makes early detection even more important with more infectious variants and means that the results of this study are likely to be even more pronounced than were estimated at the time. Reduced vaccine efficacy would be likely to increase the number of incursions and observed transmissibility, further accentuating this effect. The sensitivity analyses for TTS compliance (Figure 5) and screening frequency (Supplementary material) are equivalent to varying the test sensitivity, and suggest that a moderate reduction in RAT test sensitivity associated with new variants would be unlikely to qualitatively change our findings.

Specific to schools, limited network-type data on contact patterns within schools mean that mixing is approximated as consisting of classroom and non-classroom contacts, where students are allocated at random to classrooms and then randomly mix with other students outside of classrooms (rather than having social clustering). Some findings are also sensitive to assumptions for the number of non-classroom contacts students have; quarantine or test-to-stay strategies in particular focus on classroom contacts rather than close contacts as they are more practical to identify. However, these strategies will be less effective if a greater proportion of risk comes from non-class contacts.

Surveillance of teachers was found to have minimal benefit for reducing outbreaks in schools. Teachers only comprise a small proportion of the school community and for the purpose of this analysis we assumed that students and teachers had the same probability of becoming infected outside the school and causing the incursion. If teachers have a higher risk of becoming infected in the community than students, which may be the case (25), then the benefit of screening teachers would be higher than estimated.

Schools are embedded within their broader communities and receive incursions from the community as well as seeding cases back into the community. For this study, outbreaks were projected after a random initial incursion, without modelling the process by which the incursions occur. However, there may be social or other factors that make teachers or older/younger students more likely to be exposed in the community, and hence more likely to be the index case within the school, and these could change the effectiveness of different control measures. Limiting the scope of analysis to the outbreak within a school also meant that the benefits of community public health responses on reducing incursions into schools are not modelled, nor the benefits of school closures on reducing overall community transmission. Future work could assess the impact of community interventions on schools, and impact of school interventions on the rest of the community.

We have examined test-to-stay and quarantine protocols in the specific context of COVID-19 outbreaks, but the same questions are relevant for other respiratory infections such as influenza. The general principle of test-to-stay providing similar protection to quarantine is strongly dependent on test sensitivity, but is likely to also depend on disease attributes such as the incubation period, pre-symptomatic infectiousness, and whether there are asymptomatic carriers. Future work could investigate how such factors affect the impact of policy responses.

## Conclusion

Twice-weekly surveillance testing of students with RATs can markedly reduce outbreak risk in schools by enabling early detection of incursions and is likely to have greatest benefit in areas with higher community transmission. Following an outbreak in a school, as an alternative to home quarantine a ‘test-to-stay’ strategy for class contacts achieves equivalent outbreak containment and enables face-to-face teaching. Evaluation of both approaches in schools will be critical to inform ongoing policy decisions and to optimize implementation of testing in educational settings when needed to reduce incursions.

## Data availability statement

The original contributions presented in the study are included in the article/Supplementary material, further inquiries can be directed to the corresponding author.

## Author contributions

RA, JM, and NS conceived the study and developed the methodology. JM and NS conducted stakeholder consultations. RA, FR, MD, MH, JM, and NS devised scenarios. RA, RS-D, KH, DD, and NS developed the model. RA and NS conducted the analyses and drafted the manuscript. FR, MD, MH, and JM validated inputs and outputs. All authors contributed to the article and approved the submitted version.

## Funding

RA, RS-D, KH, DD, and NS have received funding from the Victorian Department of Health (DoH), NSW DoH and the Federal Government for modelling related to COVID-19. FMR received funding from the Victorian Government’s Department of Health and Human Services to analyze SARS-CoV-2 school outbreak data in 2020 and is a member of the Australia government’s Department of Foreign Affairs and Trade Expert Advisory Group for Regional Vaccine Access and Health Security Initiative. MD received funding from the Victorian Government’s Department of Health and Human Services to undertake enhanced public health investigation of SARS-CoV-2 cases in Victorian schools and early childhood education and care, was a member of the COVID-19 ATAGI working group on vaccine safety and confidence 2020–21 and is a member of the Australian Expert Technical Assistance Program for Regional COVID-19 Vaccine Access: Policy, Planning and Implementation (AETAP-PPI) Advisory Group. MH receives funding from the Victorian Government (DoH, Department of Families, Fairness and Housing, and Department of Jobs, Precincts and Regions) and the Macquarie Foundation to undertake work monitoring the impact of COVID-19 on the community. JM is an invited expert member of the Australian Health Protection Principal Committee, the Communicable Diseases Network of Australia COVID-19 working group and the Australian Technical Advisory Group on Immunization COVID-19 vaccine technical working group. This work was directly funded by the Australian Government Department of Health Office of Health Protection.

## Conflict of interest

The authors declare that the research was conducted in the absence of any commercial or financial relationships that could be construed as a potential conflict of interest.

## Publisher’s note

All claims expressed in this article are solely those of the authors and do not necessarily represent those of their affiliated organizations, or those of the publisher, the editors and the reviewers. Any product that may be evaluated in this article, or claim that may be made by its manufacturer, is not guaranteed or endorsed by the publisher.
