# Supplementary material for: Keeping kids in school: modelling school-based testing and quarantine strategies during the COVID-19 pandemic in Australia
Source: Front Public Health. 2023 Jun 2;11:1150810. doi: 10.3389/fpubh.2023.1150810 (PMC10272722; doi:10.3389/fpubh.2023.1150810)
Supplement: Supplementary file 1 [file Data_Sheet_1.pdf]

Keeping kids in school: modelling school-based testing and quarantine strategies during the COVID-19 pandemic in Australia

Supplementary material

Appendix A: Combined screening and test-to-stay

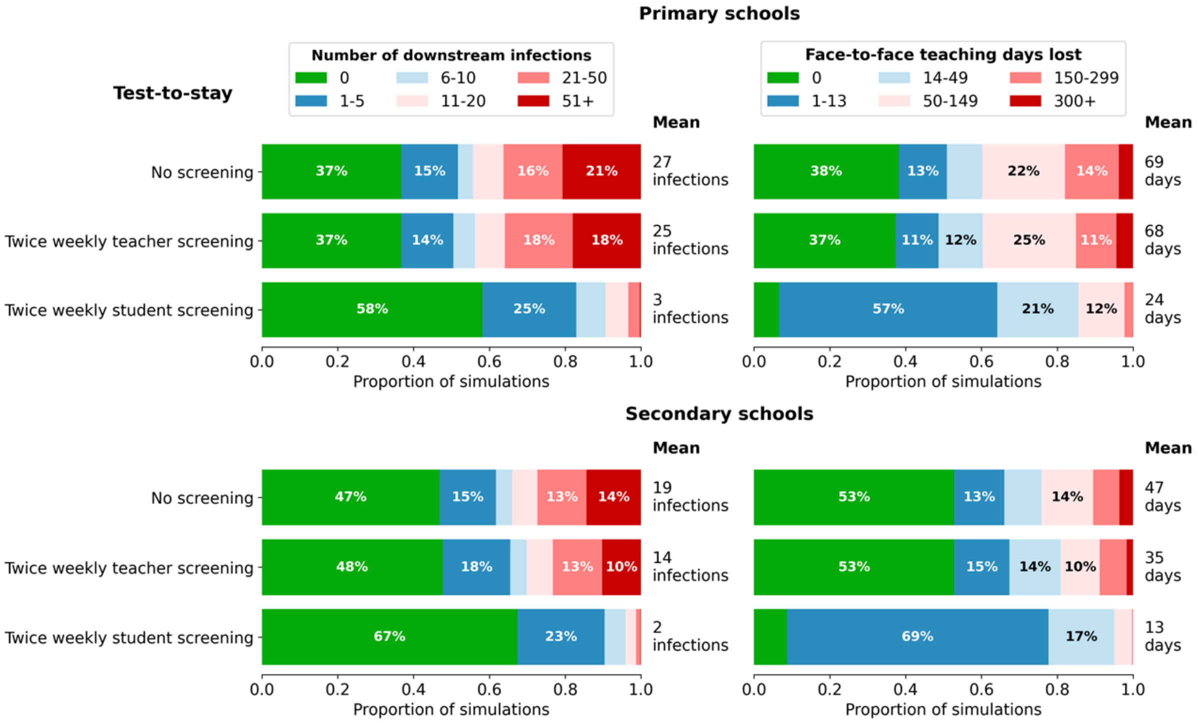

Figure S1: Impact of surveillance strategies on the distribution of outcomes for cumulative infections (left) and days of face-to-face teaching lost (right) in a single school following a single incursion. Outcomes are from 1000 model simulations run for 45 days following first diagnosis. Scenarios assume classroom contacts test-to-stay and from top to bottom are based on: no screening; twice weekly testing of teachers with rapid antigen tests; twice weekly testing of students with rapid antigen tests.

Appendix B: Total days of face-to-face teaching gained

The outputs shown above relate to the number of face-to-face teaching days lost following a single incursion. However, schools will experience ongoing incursions, with an incursion rate influenced by transmission in the surrounding community. In the absence of empirical data on the incursion rate at the time of analysis, we developed a simple method to outline how a cost-effectiveness analysis for screening may be performed, applied to a range of different community infection rates.

For a particular community, the total days of face-to-face teaching gained could be simplistically estimated by multiplying:

a) New daily cases in local community (diagnoses/day not in quarantine)
b) Proportion of new cases that occur in school-age children
c) School attendance in the community (a mixture of enrolment rates and any other community restrictions modifying attendance)
d) Screening period (days) of testing in schools (e.g., to estimate the potential impact of a term of screening)
e) Average days of face-to-face teaching gained from a single incursion in a single school due to screening (i.e., difference in average model outputs from (Figure S1).

As an example, between June and October 2021 in NSW and Victoria approximately 30% of new diagnoses occurred in people aged 18 and under (noting that 12-15-year-olds only became eligible for vaccination in September). From Figure S1, there were an average of 45 days of face-to-face teaching gained from a single incursion in primary schools, and 34 days gained in secondary schools. Using these values and varying the remaining quantities, the number of days of face-to-face teaching days gained due to screening have been estimated for a population of 100,000 over a 45-day period (Figure S2). The greatest number of face-to-face teaching days gained through screening occur when incidence is highest.

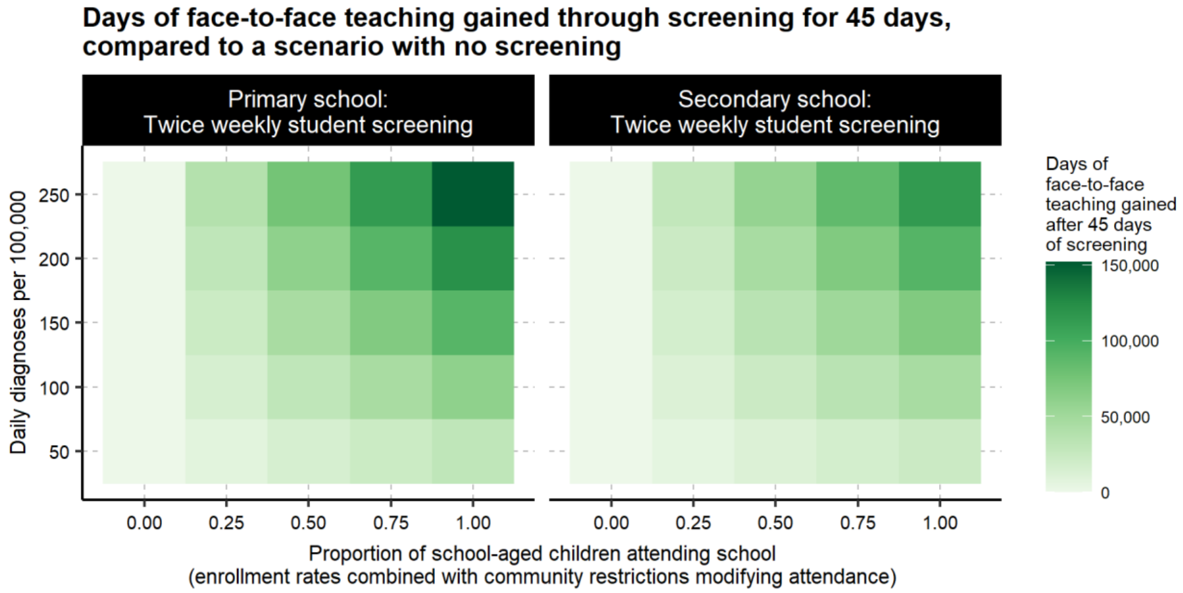

Figure S2: Estimated total days of face-to-face teaching gained through twice weekly student screening for 45 days of screening in a community with 100,000 population. Example assumes test-to-stay is in place alongside the screening. Left: Primary school. Right: secondary school. Assumes 30% of community infections are in school-aged children, an average of 45 and 34 days of face-to-face teaching are gained per incursion in primary and secondary schools respectively. Outcomes are shown for a range of community infection rates and school attendance rates (percentage of school-aged children attending school).

There are some caveats to this approach. Most notably, the proportion of the total cases occurring in school children is likely to change over time, which could considerably affect the overall estimate. There is also uncertainty in the percentage of all infections that are diagnosed, which depends on community testing rates – this is likely to underestimate incursion rates. Conversely, for communities with high transmission and frequent incursions, the outcomes of each incursion may not be independent and so this may overestimate the face-to-face teaching days gained.

Appendix C: Sensitivity analyses

We examined sensitivity of our findings to several key model parameters, in addition to those discussed in the main text.

Non-pharmaceutical Interventions in schools

Schools have implemented a range of non-pharmaceutical interventions (NPIs) to reduce transmission, such as improving ventilation/air filtration, physical distancing measures, and masks. There is a wide range of policy settings and potential impact levels, so we examined the impact of NPIs collectively. The impact of non-pharmaceutical interventions (NPIs; e.g. masks, ventilation) were tested by running scenarios where the risk of transmission per contact was reduced by either 25% or 50%. NPIs can reduce outbreak risks in schools and reduce the number of days of face-to-face teaching lost.

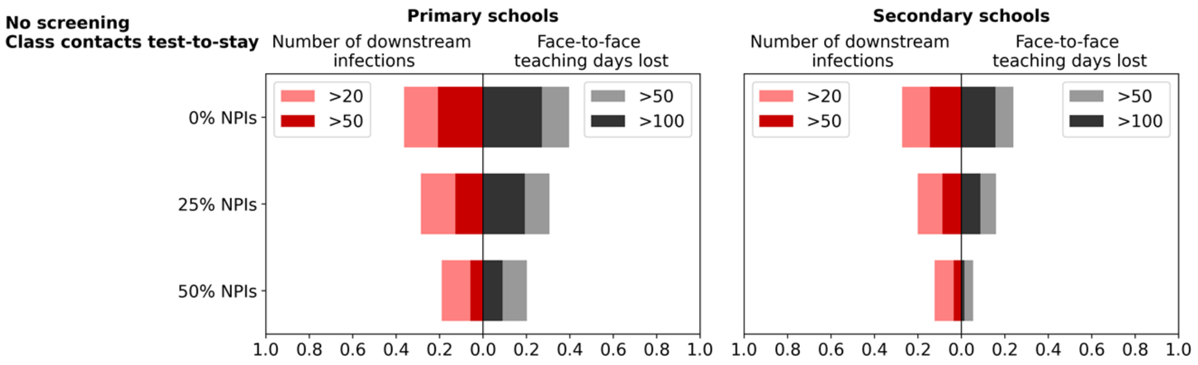

Figure S3: Impact of non-pharmaceutical interventions (NPIs) on outbreaks in schools. Red bars: the percentage of simulations with more than 20 or 50 cumulative infections after 45 days of first diagnosis. Grey bars: the percentage of simulations with more than 50 or 100 days of face-to-face teaching lost in a single school following an incursion. Scenarios assume test-to-stay is in place for class contacts and no surveillance testing.

Vaccine coverage in students

We varied vaccine coverage to account for uncertainty in child vaccine uptake, as well as to understand the contribution of existing vaccines in secondary schools. The vaccine coverage levels examined were:

- 0%, 60%, 80% coverage among students 12+ years
- 0%, 60%, 80% coverage among students 5-11 years

As shown in Figure S4, the vaccines strongly impact both outbreak size and days of face-to-face teaching lost.

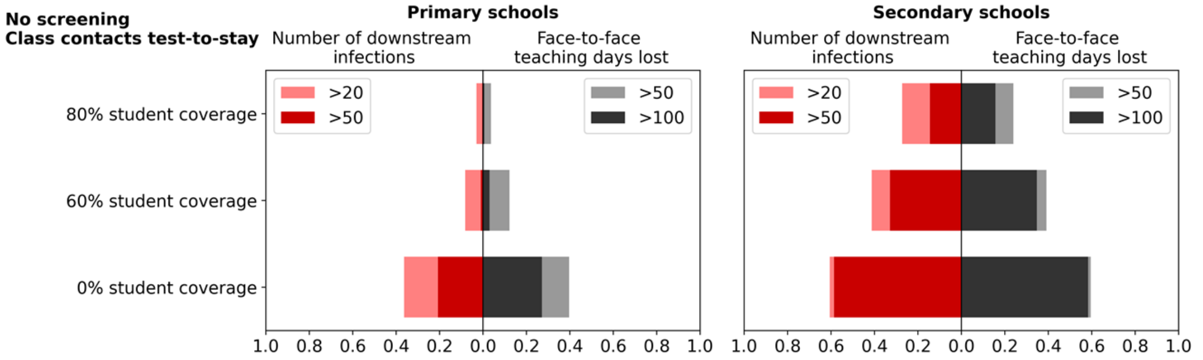

Figure S4: Impact of vaccines for students on outbreaks in schools. Red bars: the percentage of simulations with more than 20 or 50 cumulative infections after 45 days of first diagnosis. Grey bars: the percentage of simulations with more than 50 or 100 days of face-to-face teaching lost in a single school following an incursion. Scenarios assume test-to-stay is in place for class contacts and no surveillance testing.

Vaccine coverage in teachers

We also examined the impact of vaccines among teachers, simulating coverage levels of 60%, 80%, and 100%. We note that the benefits of vaccinating teachers are not fully captured in this analysis, since the model does not account for potential reduced incursions as a result of teacher vaccination – only reduced transmission within the school once an incursion has already occurred.

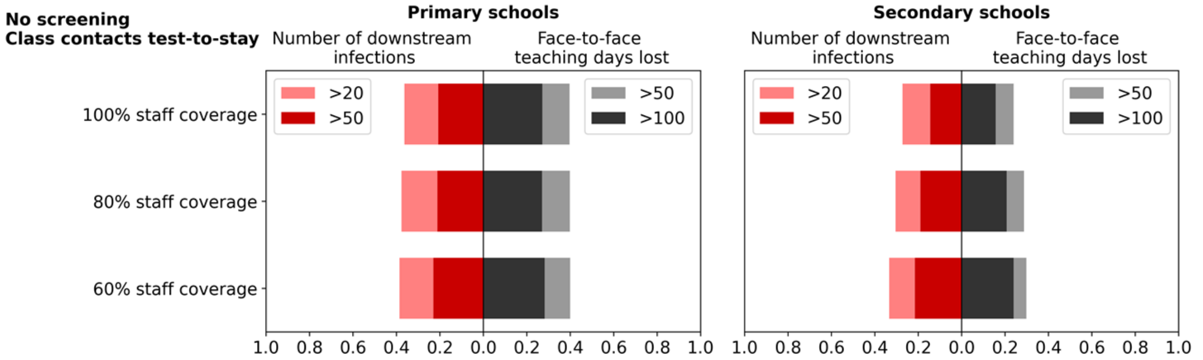

Figure S5: Impact of vaccines for teachers on outbreaks in schools. Red bars: the percentage of simulations with more than 20 or 50 cumulative infections after 45 days of first diagnosis. Grey bars: the percentage of simulations with more than 50 or 100 days of face-to-face teaching lost in a single school following an incursion. Scenarios assume test-to-stay is in place for class contacts and no surveillance testing.

Frequency of surveillance screening

The baseline scenarios examined twice-weekly surveillance testing. We examined daily surveillance screening to assess the extent to which more frequent screening could further improve containment, and weekly screening, to explore options for decreasing costs or the impact of reduced compliance. As shown in Figure S6, twice-weekly screening was able to achieve most of the benefits on decreasing infections, with diminishing returns from increasing testing frequency. However, further gains in reducing days of teaching lost could be achieved by more frequent screening.

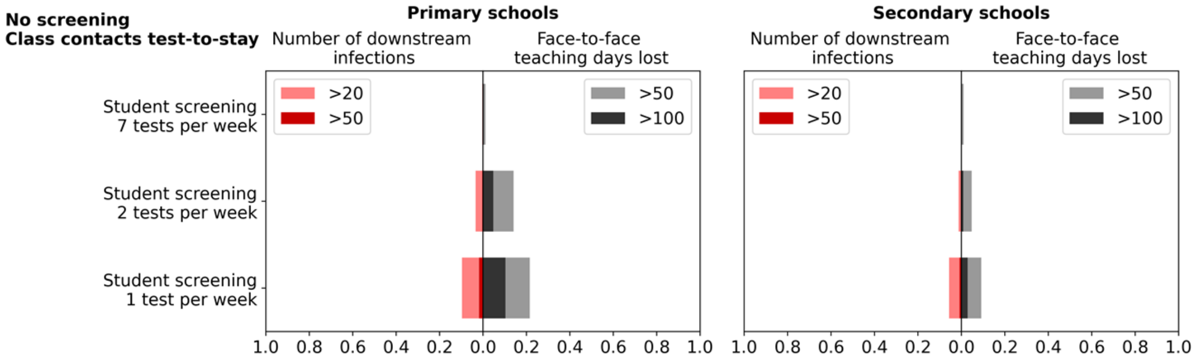

Figure S6: Impact of different frequencies of surveillance testing on outbreaks in schools. Red bars: the percentage of simulations with more than 20 or 50 cumulative infections after 45 days of first diagnosis. Grey bars: the percentage of simulations with more than 50 or 100 days of face-to-face teaching lost in a single school following an incursion. Scenarios assume test-to-stay is in place for class contacts.

Symptomatic testing rate

The model has an underlying parameter for the per-day probability that an individual with mild COVID-19 symptoms will have a test. This parameter plays an important role in determining how long it takes to detect an outbreak in scenarios where regular testing of students or teachers are not in place. Hence a sensitivity analysis was run to understand what influence this parameter had on key outcomes. Figure S7 shows that maintaining symptomatic testing is important for earlier detection of outbreaks and reduced outbreak size.

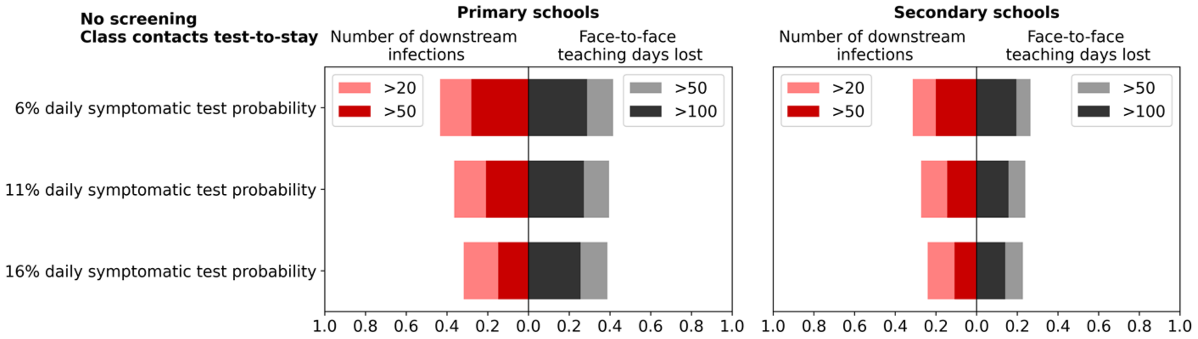

Figure S7: Impact of symptomatic testing probability on outbreaks in schools. Red bars: the percentage of simulations with more than 20 or 50 cumulative infections after 45 days of first diagnosis. Grey bars: the percentage of simulations with more than 50 or 100 days of face-to-face teaching lost in a single school following an incursion. Scenarios assume test-to-stay is in place for class contacts and no surveillance testing.

Sensitivity to number of non-classroom contacts

As discussed in the main text, containment policies are largely targeted at classroom contacts, but the extent to which they impact transmission within schools depends on how much mixing there is across classrooms. We examined sensitivity to the average number of non-classroom contacts per student, to account for uncertainty in mixing behaviour outside classrooms in schools (e.g., in playgrounds, pick up/drop off, sports etc.). Figure S8 shows that although the benefit of applying test-to-stay to entire schools rather than classrooms is greater when there is more cross-classroom mixing (as expected), the effect is relatively weak, for the parameter values examined here.

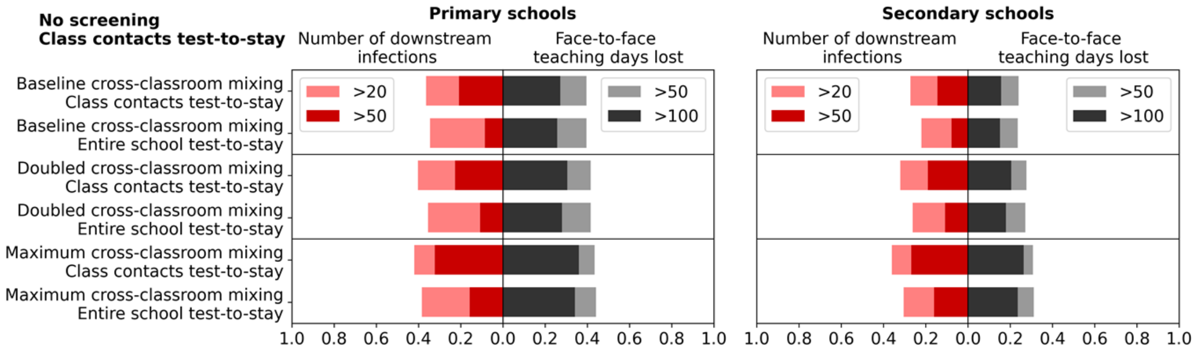

Figure S8: Impact of assumptions around number of non-classroom contacts per student. Doubled cross-classroom mixing assumes 4 and 10 non-classroom contacts for primary and secondary school students respectively. Maximum cross-classroom mixing assumes 11 and 22 non-classroom contacts for primary and secondary school students respectively. Red bars: the percentage of simulations with more than 20 or 50 cumulative infections after 45 days of first diagnosis. Grey bars: the percentage of simulations with more than 50 or 100 days of face-to-face teaching lost in a single school following an incursion. Scenarios assume no surveillance testing.

Appendix D: Additional methodological details

The agent-based model Covasim models the spread of COVID-19 by simulating a collection of agents representing people. Each agent is characterised by a set of demographic and disease properties:

- Demographics:
    - Age (one-year brackets)
    - Household size, and uniquely identified household members
    - Uniquely identified school contacts (for people aged 5-18)
    - Uniquely identified work contacts (for people aged 18-65)
    - Average number of daily community contacts (multiple settings / contact networks modelled, described below)
- Disease properties:
    - Infection status (susceptible, exposed, recovered or dead)
    - Whether they are infectious (no, yes)
    - Whether they are symptomatic (no, mild, severe, critical; with probability of being symptomatic increasing with age, and the probability of symptoms being more severe increasing with age)
    - Diagnostic status (untested vs tested)

Transmission is modelled to occur when a susceptible individual is in contact with an infectious individual through one of their contact networks. The probability of transmission per contact is calibrated to match the epidemic dynamics observed and is weighted according to whether the infectious individual has symptoms, and the type of contact (e.g. household contacts are more likely to result in transmission than community contacts). Transmission dynamics depend on the structure of these contact networks, which are randomly generated but statistically resemble the specific setting being modelled. The layers included are described below, and the model parameters values are provided for each layer that was included.

Model population

For this analysis a synthetic model population was initialized comprising of 100,000 people. The age and household size structure of the model population was based on the Australian population.

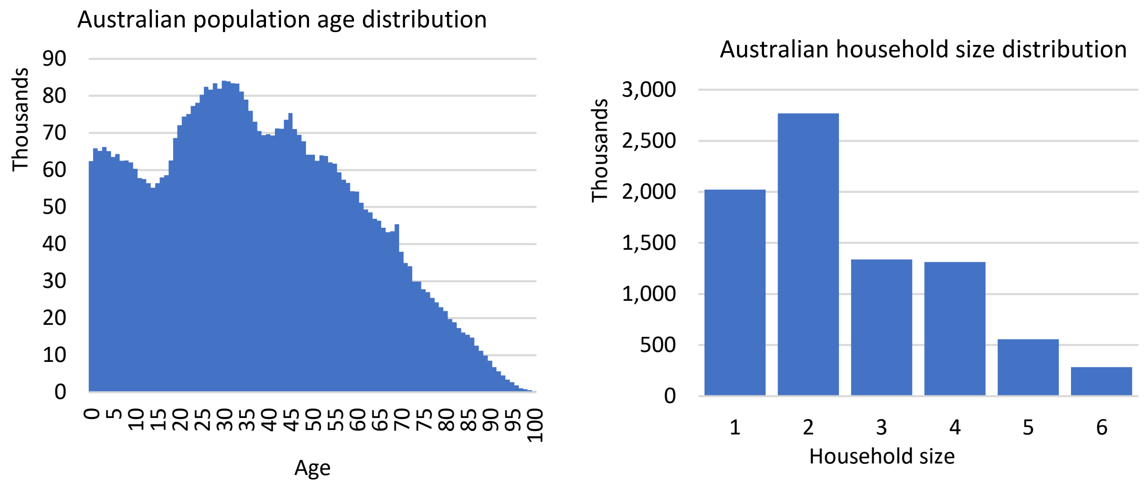

Figure S9: Population age structure and household size distribution [1].

Household contact network: household size and age structure

The household contact network was set up by explicitly modelling households. The households size distribution for Australia [1] was scaled to the number required for the number of agents in the simulation. Each person in the model was uniquely allocated to a household. To assign ages, a single person was selected from each household as an index, whose age was randomly sampled from the distribution of ages of the Household Reference Person Indicator in the 2016 Census [1]. The age of additional household members were then assigned according to Australian age-specific household contact estimates from Prem et al. [2], by drawing the age of the remaining members from a probability distribution based on the row corresponding to the age of the index member.

School contact networks

Schools and school contact networks were set up as described in the main report.

Work contact networks

Two different workplace types are included: public facing (e.g. retail, hospitality) and non-public facing. Contact networks for non-public facing workplaces were created as a collection of disjoint, completely connected clusters for the percentage of people aged 18-65 who worked in those settings. The mean size of each cluster was equal to the estimated average number of daily work contacts (Table S1). For the percentage of people aged 18-65 who worked in public facing workplaces, their workplace networks consisted of a completely connected cluster with other work colleagues, as well as each day having a number of random contacts with the community.

Additional contact networks

An arbitrary number of additional networks can be added. Each network layer requires inputs for: the proportion of the population who undertake these activities; the average number of contacts per day

associated with these activities; the risk of transmission relative to a household contact (scaled to account for (in)frequency of some activities such as pubs/bars once per week); relevant age range; type of network structure (random, clustered, or specialized [as per schools/workplaces]); and effectiveness of quarantine and contact tracing interventions. Parameters for the networks currently in the model are in Tables S1 and S2.

Parameter values for each contact network

Tables S1 and S2 show the parameters that define each contact network in the model. Unless otherwise noted, parameters are derived in [3] from a mix of published and grey literature and a Delphi parameter estimation process. The columns refer to:

- **Network structure type:** Clustered refers to a network structure comprised of disjoint, completely connected groups of contacts. Random refers to individuals being allocated connections to anyone else in the network. Random networks are also dynamic and regenerated each day. Public facing networks are a combination of completely connected clusters for staff, who are then connected to random community members
- **Mean contacts:** The average number of contacts per person in each network. Each person in the model has their individual number of contacts draw at random from a Poisson distribution with these values as the mean. For the social network layer, a negative binomial distribution was used with dispersion parameter 2 to account for a longer tail to the distribution.
- **Mean public-public contacts:** For the percentage of people who participate in an activity, the average number of contacts they have with other members of the public (draw at random from a Poisson distribution with these values as the mean)
- **Mean public-staff contacts:** For the percentage of people who participate in an activity, the average number of contacts they have with staff (draw at random from a Poisson distribution with these values as the mean)
- **Relative transmission risk:** The transmission probability per contact is expressed relative to household contacts, and reflects the risk of transmission depending on behaviour. For example, a casual contact in a public park is less likely to result in a transmission event compared to a contact on public transport. Similarly, the relative transmission risks between staff-staff, public-public and staff-public are characterised for public-facing workplaces.
- **Quarantine effect:** If a person is quarantined, the transmission probability is reduced by this factor. For example, an individual on quarantine at home would likely not work or use public transport, but they may still maintain their household contacts.
- **Population proportion:** Each network will only include a subset of the population e.g. every person has a household, but not every person regularly uses public transport.
- **Age bound:** Each network will only include agents whose age is within this range.
- **Contact tracing probability:** Probability that each contact can be notified in order to quarantine
- **Effectiveness of quarantine and isolation:** When a close contact is asked to quarantine for 14 days, or a confirmed case asked to isolate while they are infected, these parameters represent he effectiveness of at reducing transmission through the specific networks. For example quarantine is assumed to have no impact on household transmission and greater impact on other contacts, reflecting compliance.

Table S1: Contact parameters for each of the networks in the model.

Contact network	Network structure type*	Mean contacts	Mean public-public contacts	Mean public-staff contacts	% of workforce	Relative transmission risk	Relative transmission risk (staff-staff)	Relative transmission risk (public-public)	Relative transmission risk (staff-public)	% of population	Age bound
House	Specialized	4				1.00					
School	Specialized										5-17
Non-retail work	Specialized	5			0.80	0.28					
Retail work	Public facing	5	8	2	0.11		0.28	0.04	0.04	0.70	12+
Community (general)	Random	1				0.10				1.00	
Places of worship	Clustered	20				0.04				0.11	
Community sport	Clustered	30				0.07				0.34	4-30
Entertainment	Public facing	25	8	2	0.02		0.28	0.01	0.01	0.30	15+
Cafe/restaurant	Public facing	5	8	2	0.02		0.28	0.04	0.04	0.60	12+
Pub/bar	Public facing	5	8	2	0.03		0.28	0.06	0.06	0.40	18+
Public transport	Random	25				0.16				0.11	15+
Public parks	Random	10				0.03				0.60	
Child care	Clustered	20				0.25#				0.55	1-6
Social	Random	6 (disp=2)				0.12				1.00	15+
Aged care	Clustered	12				0.58				0.07	65+

Table S2: Contact tracing parameters for each of the networks in the model.

Contact network	Assumed contact tracing probability	Assumed effectiveness of quarantine on network	Assumed effectiveness of isolation on network
House	1	0.00	0.80
School	0.95	0.99	0.99
Non-retail work	0.95	0.90	0.90
Retail work	0.95	0.90	0.90
Community (general)	0.1	0.80	0.80
Places of worship	0.5	0.99	0.99
Community sport	0.5	1.00	1.00
Entertainment	0.5	1.00	1.00
Cafe/restaurant	0.5	1.00	1.00
Pub/bar	0.5	1.00	1.00
Public transport	0.1	0.99	0.99
Public parks	0.1	1.00	1.00
Child care	0.95	0.99	0.99
Social	0.75	0.50	0.80
Aged care	0.95	0.80	0.80

Contact tracing: non-school contacts

Following detection of a positive case, the model initiates a contact tracing algorithm. *For cases detected in schools, this is described in the main report*. For cases in the community, the testing/contact tracing system was approximated as follows:

1. Day 0: Test is taken by index case
2. Day 1 (24 hours following test): Positive test results are returned, index case is notified and enters isolation.
3. Day 2 (48 hours following test being taken^): Contact tracing completed, with contacts having a setting-specific probability of being detected (Table S2), reflecting differences in the level of difficult in identifying contacts in that network (e.g. households vs public transport contacts). Identified contacts are tested and quarantined for 14 days regardless of test results, along with their entire households. Contacts are additionally tested on day 11 of quarantine, regardless of symptoms.
4. Day 3 (72 hours following test): Test results for contacts become available, and any contacts who returned a positive initial test would then have their contacts traced within the next 24 hours, in the same manner as the index case.

It was assumed that contact tracing deteriorated as case numbers increased. Caps on contact tracing assumed: at 0, 25, 75, 150 and 500+ cases per day, 100%, 80%, 50%, 30% or 20% of detected cases are subject to the above algorithm. The cap does not apply to household, school or childcare contacts who are assumed able to conduct their own tracing.

Virus strain

The model was based on transmission of the delta variant, with infectiousness calibrated to outcomes of the 2021 Victorian epidemic wave. The incubation period was shortened to a mean time from exposure to becoming infectious of 3.71 days, compared to 4.50 days for the wild type virus [4].

Vaccine properties

In the model, vaccination acts to reduce the probability of acquiring an infection when a contact occurs with an infectious case, as well as the probability of developing symptoms (both mild and severe) for people who are vaccinated and become infected. The assumed efficacy values used in this modelling are as per the main report and are provided in Table S3 [5, 6].

Table S3: Vaccine efficacy parameters.

**Vaccine**	**Reduction in infection**	**Reduction in onward transmission**
Astra-Zeneca (2 doses)	67%	36%
Pfizer (2 doses)	80%	65%

The vaccine's prevention of infection is approximated as "leaky", meaning that each person vaccinated has reduced but non-zero risk of becoming infected based on the vaccine efficacy (as opposed to an "all or nothing" vaccine, where 80% efficacy means that 80% of people have perfect protection and 20% have no protection).

Model calibration

Model parameters for transmission and testing were calibrated to data on daily new detected cases, hospitalisations and ICU from the delta COVID-19 epidemic wave in Melbourne over the July-September 2021 period [7]. The model was initialised with a population of 100,000 agents, and the overall transmission risk per contact (which multiplies the transmission probabilities in Table S1 for each layer), the per-day probability of a symptomatic individual seeking testing were varied such that the distribution of model outcomes for diagnoses, hospitalizations and number of tests was centred near the actual epidemic trajectory. For additional details see [7].

For this analysis, the model was initialized with only a single case in a school, as described in the main report, however the transmission and testing parameters were based on this previous calibration.

Supplement references

1. Australian Bureau of Statistics (ABS). Australian Bureau of Statistics 2016, Census of Population and Housing, TableBuilder. Findings based on use of ABS TableBuilder data. https://www.abs.gov.au/ausstats/abs@.nsf/web+pages/Citing+ABS+Sources#TableBuilder i. 2020.
2. Prem K, Cook AR, Jit M. Projecting social contact matrices in 152 countries using contact surveys and demographic data. PLoS computational biology. 2017;13(9):e1005697.
3. Scott N, Palmer A, Delport D, Abeysuriya R, Stuart R, Kerr CC, et al. Modelling the impact of relaxing COVID-19 control measures during a period of low viral transmission. Med J Aust. 2021;214(2):79-83.
4. Li B, Deng A, Li K, Hu Y, Li Z, Xiong Q, et al. Viral infection and transmission in a large well-traced outbreak caused by the Delta SARS-CoV-2 variant. MedRxiv. 2021.
5. Pouwels KB, Pritchard E, Matthews PC, Stoesser N, Eyre DW, Vihta K-D, et al. Effect of Delta variant on viral burden and vaccine effectiveness against new SARS-CoV-2 infections in the UK. Nature Medicine. 2021;27(12):2127-35. doi: 10.1038/s41591-021-01548-7.
6. Eyre DW, Taylor D, Purver M, Chapman D, Fowler T, Pouwels KB, et al. Effect of Covid-19 Vaccination on Transmission of Alpha and Delta Variants. New England Journal of Medicine. 2022;386(8):744-56. doi: 10.1056/nejmoa2116597.
7. Abeysuriya R, Delport D, Sacks-Davis R, Hellard M, Scott N. Modelling the Victorian roadmap. 18 September 2021. Available from: https://www.burnet.edu.au/system/asset/file/4942/Burnet_Institute_VIC_Roadmap_20210918_-FINAL.pdf. [Public Health Report]. 2021.
